# Increased Neurofilament Light Chain and YKL-40 CSF Levels in One Japanese IBMPFD Patient With VCP R155C Mutation: A Clinical Case Report With CSF Biomarker Analyses

**DOI:** 10.3389/fneur.2020.00757

**Published:** 2020-08-11

**Authors:** Masaki Ikeda, Takeo Kuwabara, Eriko Takai, Hiroo Kasahara, Minori Furuta, Akiko Sekine, Kouki Makioka, Tsuneo Yamazaki, Yukio Fujita, Kazuaki Nagashima, Tetsuya Higuchi, Yoshito Tsushima, Yoshio Ikeda

**Affiliations:** ^1^Department of Neurology, Gunma University Graduate School of Medicine, Maebashi, Japan; ^2^Department of Neurology, Jobu Hospital for Respiratory Diseases, Maebashi, Japan; ^3^Department of Occupational Therapy, Gunma University Graduate School of Health Sciences, Maebashi, Japan; ^4^Department of Diagnostic Radiology and Nuclear Medicine, Gunma University Graduate School of Medicine, Maebashi, Japan

**Keywords:** IBMPFD, *VCP*, mutation, CSF, NFL, YKL-40, AD, frontotemporal dementia

## Abstract

Inclusion body myopathy (IBM) with Paget's disease of bone (PDB) and frontotemporal dementia (IBMPFD) presents with multiple symptoms and an unknown etiology. Valosin-containing protein (*VCP*) has been identified as the main causative gene of IBMPFD. However, no studies on neurofilament light chain (NFL) as a cerebrospinal fluid (CSF) marker of axonal neurodegeneration or on YKL-40 as a CSF marker of glial neuroinflammation have been conducted in IBMPFD patients with *VCP* mutations. A 65-year-old man presented with progressive muscle atrophy and weakness of all limbs, non-fluent aphasia, and changes in personality and behavior. Cerebral MRI revealed bilateral frontal and temporal atrophy. ^99m^Tc-HMDP bone scintigraphy and pelvic CT revealed remodeling changes and active osteoblastic accumulations in the right medial iliac bone. Muscle biopsy demonstrated multiple rimmed vacuoles in muscle cells with myogenic and neurogenic pathological alterations. After the patient was clinically diagnosed with IBMPFD, DNA analysis of the *VCP* gene revealed a cytosine (C) to thymine (T) (C→ T) mutation, resulting in an amino acid exchange of arginine to cysteine (p.R155C mutation). The CSF levels of NFL at two time points (12 years apart) were higher than those in non-dementia controls (CTR) and Alzheimer's disease (AD); lower than those in frontotemporal dementia with motor neuron disease (FTD-MND); and comparable to those in patients with behavioral variant frontotemporal dementia (bvFTD), progressive supranuclear palsy (PSP), and corticobasal syndrome (CBS). The CSF levels of YKL-40 were comparable at both time points and higher than those in CTR; lower than those in FTD-MND; and comparable to those in bvFTD, PSP, CBS, and AD. The CSF levels of phosphorylated tau 181 (P-Tau) and total tau (T-Tau) were not significantly different from those in CTR and other neurodegenerative diseases, except those in AD, which were significantly elevated. This is the first report that demonstrates increased NFL and YKL-40 CSF levels in an IBMPFD patient with a *VCP* mutation (p.R155C); NFL and YKL-40 levels were comparable to those in bvFTD, PSP, CBS, and AD and higher than those in CTR. Our results suggest that IBMPFD neuropathology may involve both axonal neurodegeneration and glial neuroinflammation.

## Introduction

Inclusion body myopathy (IBM) with Paget's disease of bone (PDB) and frontotemporal dementia (IBMPFD) is a multi-organ disease with still unknown etiology (1, 2). In IBMPFD with autosomal dominant inheritance, valosin-containing protein (*VCP*) has been identified as the major causative gene (2, 3). Neurofilament light chain (NFL), which is indicative of axonal neurodegeneration (4), has been validated as a CSF biomarker of behavioral variant frontotemporal dementia (bvFTD), FTD with motor neuron disease (FTD-MND), amyotrophic lateral sclerosis (ALS), progressive supranuclear palsy (PSP), corticobasal syndrome (CBS), and Alzheimer's disease (AD) (5–7). Furthermore, YKL-40 (known as chitinase 3-like 1) has been reported as a CSF biomarker of glial neuroinflammation in neurodegenerative diseases (6–8). With regard to IBMPFD with *VCP* mutations, no CSF studies on NFL or YKL-40 have been conducted yet.

## Case Presentation

We describe the case of a 65-year-old man who presented with muscle weakness and atrophy of all limbs. At the age of 42 years, he experienced difficulties in standing from a sitting position and raising his arms over his head. At the age of 48 years, he was affected by gait disturbances with difficulties squatting and was able to walk only at a slow pace. Further, the patient could not raise his arms over his head and experienced difficulties moving his head and neck freely. These symptoms gradually deteriorated. During the first hospitalization at the age of 52 years, the patient showed atrophy and weakness of the muscles of all limbs but most prominently of the bilateral quadriceps. The neuropsychological examination revealed decline in his cognitive function. The scores of the Mini-Mental State Examination (MMSE) and the Montreal Cognitive Assessment (MoCA) were 26/30 and 18/30, respectively, with disturbances of attention and executive functions. The score of the frontal assessment battery (FAB) was 8/18 with disturbances of “similarities,” “lexical fluency,” and “motor series.” However, no remarkable changes of character, behavior, voice, and speech were observed. The patient showed generalized hyporeflexia without pathologic reflexes. He exhibited no respiratory difficulty. The CT showed prominent atrophy of the quadriceps and other muscles, e.g., hamstrings, iliopsoas, and anterior tibial muscles (not shown). Because of gait difficulty due to weakness of the legs, the patient used a cane or a walker at the age of 52 years (after the first hospitalization), and he used a wheelchair at the age of 55 years. He had occasional cough due to dysphasia and difficulty expectorating, when he was 60 years old; at the same time, he exhibited character changes including self-centered thinking, extreme dependence on his wife, irritation, and frustration. Furthermore, the patient rejected or was indifferent to advice from others. At the age of 61 years, he frequently coughed and experienced shortness of breath due to saliva and food; subsequently, he suffered from dysphagic pneumonia due to massive saliva and was finally readmitted to our hospital.

During the second hospitalization, the muscles of the patient's four limbs revealed more pronounced weakness and atrophy than during the first hospitalization. Generalized hyporeflexia was still present; however, bilateral Babinski reflexes were observed. A neuropsychological examination was conducted, when the patient improved after the pneumonia. The MMSE score was 21/30, whereas the MoCA score was 12/30 with disturbed attention, visuospatial cognition, and executive functions. The FAB score was 6/18 with disturbances of “similarities,” “lexical fluency,” “motor series,” and “prehension behavior.” The results of the neuropsychological tests revealed a deterioration of cognitive functions including mainly language and speech disturbances due to predominantly frontal and temporal lobe dysfunctions. His speech was apparently affected by non-fluent agrammatic primary progressive aphasia (naPPA) with word-finding difficulties and mistakes of words and characters. The changes in personality presented as adhesion, irritation, dependent tendencies, and self-centered behavior with childish manners. After the pneumonia improved, the patient was moved to another hospital, and his treatment continued. The patient was alert and could speak with the help of a speech cannula after a tracheotomy; however, he could also communicate independently with blinking. He needed frequent aspiration of saliva and oxygen inhalation to support his respiration. At the present age of 65 years, a lumbar puncture was performed, after we obtained the patient's informed consent.

**Figure 1:**
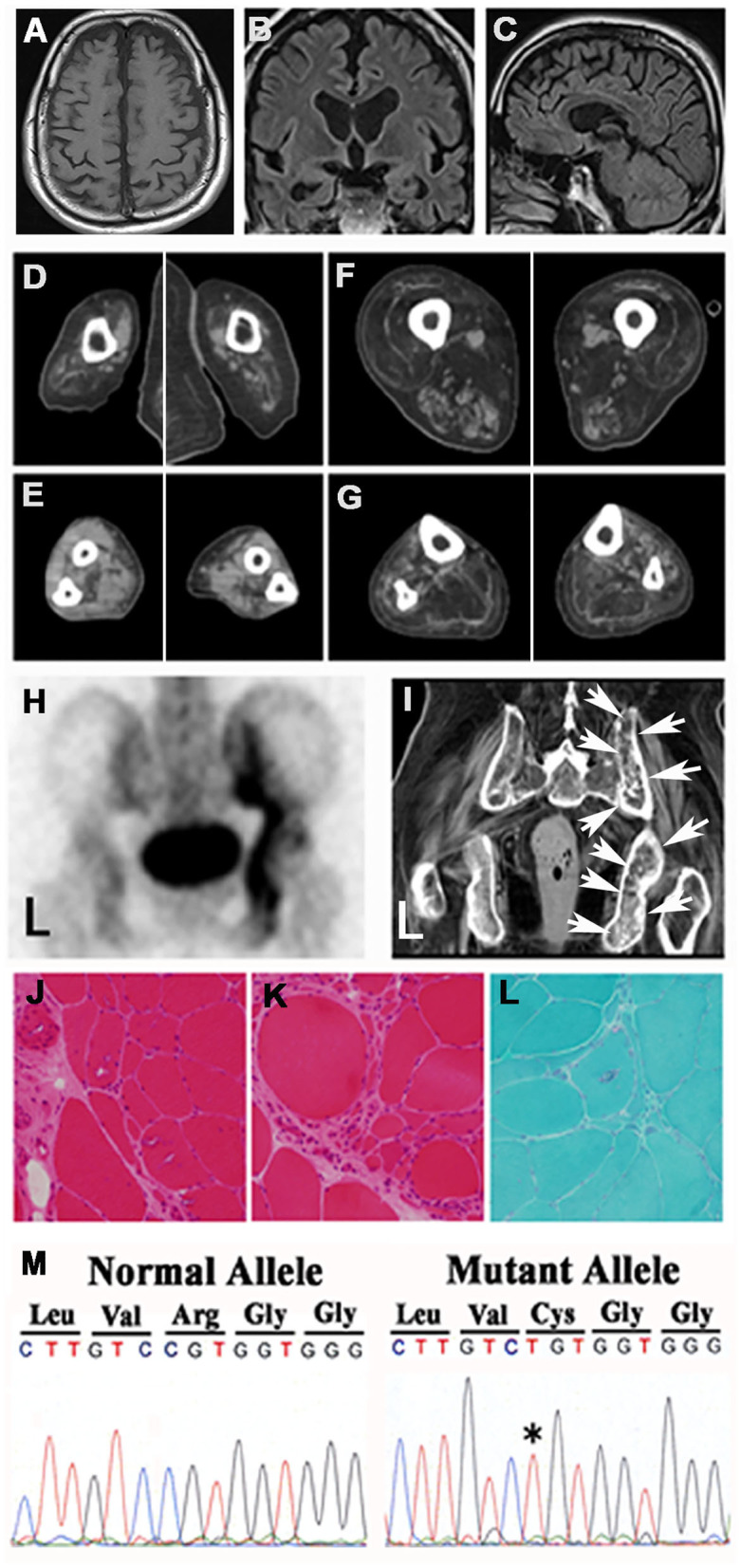
Findings of images, pathological examinations, and DNA sequences. Cerebral MRIs of the **(A)** transverse view, **(B)** coronal view, and **(C)** sagittal view demonstrated frontal and temporal lobe atrophy. CT of muscles of the **(D)** upper arms, **(E)** forearms, **(F)** thighs, and **(G)** lower legs showed muscle atrophy in the four extremities. **(H)** ^99m^Tc-HMDP bone scintigraphy of the pelvis revealed active osteoblastic accumulation in the right medial iliac bone. **(I)** Pelvic CT showed remodeling changes in the corresponding area designated with arrows **(H)**. Microscopic findings. Hematoxylin eosin staining showed multiple rimmed vacuoles in muscle cells **(J)** and numerous small fibers **(K)**. **(L)** Gomori trichrome staining demonstrated rimmed vacuoles and small angulated fibers. **(M)** Genomic DNA analysis revealed a missense mutation in the *VCP* gene that exchanged CGT (Arg) to T*GT (Cys).

The patient's mother had also shown muscular weakness and bilateral atrophy of the lower limbs at the age of 60 years, eventually also involving the upper limbs, which had resulted in her becoming bed-ridden. She was diagnosed with amyotrophic lateral sclerosis (ALS) and died from pneumonia at the age of 68 years; it was not confirmed whether she had been affected by dementia. The patient's father died from pancreatic cancer, whereas his elder sister suffered from gait disturbance of unknown etiology since her childhood and died from brain tumor at the age of 40 years. His younger brother died from malignant lymphoma at the age of 36 years. The patient did not have any children. During the first hospitalization, cerebral MRI showed bilateral frontal and temporal atrophy (Figures 1A–C). During the second hospitalization, CT of the extremities exhibited severe bilateral muscle atrophy of the upper arms, forearms, thighs, and lower legs (Figures 1D–G). During the second hospitalization, ^99m^Tc-HMDP bone scintigraphy showed active osteoblastic accumulation in the right medial iliac bone (Figure 1H), whereas pelvic CT revealed remodeling changes in the corresponding area indicated by arrows (Figure 1I). Hematoxylin and eosin staining of the muscle biopsy specimens demonstrated multiple rimmed vacuoles in muscle cells (Figure 1J) and numerous small fibers and round-shaped fibers (Figure 1K). Gomori trichrome staining showed rimmed vacuoles in muscle cells and small angulated fibers (Figure 1L), which were compatible with the pathological findings of IBMPFD during the first hospitalization. DNA analysis revealed a cytosine (C) to thymine (T) (C→T^*^) mutation, resulting in an amino acid exchange of arginine to cysteine (p.R155C) (Figure 1M) as previously described (2, 3, 9–14).

The neurological finding of this case revealed general muscle weakness and atrophy, especially, proximal muscles of lower extremities, progressive cognitive decline, speech disturbance, and character change. In muscle biopsy, rimmed vacuoles were pathologically confirmed and neurogenic muscle changes were also observed. ^99m^Tc-HMDP bone scintigraphy of the patient was compatible with Paget's disease of bone (PDB).

## Methods and Results of the CSF Analyses

The patient was examined by lumbar puncture two times (during the present hospitalization and 13 years earlier). The CSF samples obtained from the patient by the two lumbar punctures were stored separately in 1.5-ml Eppendorf tubes. The CSF samples were strictly stored in a −80°C freezer and never opened nor freeze-thawed, until they were measured using enzyme-linked immunosorbent assay (ELISA) kits. Phosphorylated Tau (P-Tau), human total tau (T-Tau), neurofilament light chain (NFL), and YKL-40 were measured. The CSF samples were analyzed in this patient, patients with neurodegenerative diseases (bvFTD: *n* = 7, FTD-MND: *n* = 5, PSP: *n* = 7, CBS: *n* = 7, and AD: *n* = 24), and non-dementia control subjects (CTR: *n* = 18). The patients with IBMPFD, bv-FTD, FTD-MND, PSP, CBS, and AD were diagnosed in accordance with the global clinical criteria [IBMPFD (1, 2, 15); bv-FTD and FTD-MND (16–21); PSP (22, 23); CBS (24); and AD (25–27)] by experienced neurologists (M.I., T.K., H.K., M.F., K.M, K.N., Y.F., and Y.I.) at the Department of Neurology, Gunma University Hospital (Table 1). ^99m^Tc-HMDP bone scintigraphy findings in IBMPFD patients were evaluated by senior radiologists (T.H. and Y.T.). P-Tau and T-Tau in CSF were analyzed with sandwich ELISA INNOTEST® PHOSPHO-TAU(181P) (Fujirebio Europe N.V., Gent, Belgium) (28, 29) and sandwich ELISA INNOTEST® T-Tau-Ag (Fujirebio Europe N.V., Gent, Belgium) (30), respectively. NFL and YKL-40 CSF levels were measured utilizing sandwich ELISA NF-light® (IBL International, Hamburg, Germany) (4–7) and MicroVue™ YKL-40 EIA kits (Quidel, San Diego, CA, USA) (7, 8), respectively.

**Table 1:** Demographic characteristics of the patients with IBMPFD and neurodegenerative diseases and of non-dementia control subjects.

	**IBMPFD**	**bvFTD**	**FTD-MND**	**PSP**	**CBS**	**AD**	**CTR**
No.	1	7	5	7	7	24	18
Male	100	71.43	40.00	42.86	57.14	45.83	50.00
Age at onset (years old)	48	52 ± 3.24	60 ± 3.22	69 ± 1.93	68 ± 2.38	64 ± 1.58	–
Age at CSF analysis	#1: 52	55 ± 2.66	62 ± 2.82	71 ± 1.90	70 ± 2.53	69 ± 1.49	65 ± 2.34
	#2: 65						
MMSE (/30)	#1: 26	14 ± 3.58	18 ± 1.59	22 ± 2.28	16 ± 3.24	20 ± 1.08	29 ± 0.25
	#2: 21						
MoCA (/30)	#1: 18	6 ± 2.76	13 ± 2.40	19.5 ±4.25	14 ± 4.87	16 ± 0.99	28.5 ± 0.36
	#2:12						
FAB (/18)	#1: 8	8.5 ± 3.80	6.5 ± 1.31	7 ± 1.03	9 ± 2.65	9.5 ± 0.67	17 ± 0.28
	#2: 6						
NFL (pg/ml)	#1: 5,255.24	5,493.71 ± 814.18	9,371.82 ± 1,134.69	4,413.78 ± 741.49	4,217.29 ± 936.81	1,531.70 ± 167.56	452.93 ± 58.90
	#2: 5,394.98						
YKL-40 (ng/ml)	#1: 125.03	146.07 ± 25.87	154.39 ± 62.41	99.94 ± 17.62	84.59 ± 17.68	107.23 ± 10.26	60.53 ± 5.43
	#2: 132.41						
P-Tau (pg/ml)	#1: 31.79	41.75 ± 6.10	31.45 ± 6.87	36.60 ± 3.61	33.93 ± 7.08	76.42 ± 7.73	25.52 ± 2.53
	#2: 34.72						
T-Tau (pg/ml)	#1: 148.60	319.93 ± 50.07	213.99 ± 68.27	231.09 ± 63.39	90.61 ± 50.96	506.86 ± 71.60	143.56 ± 16.07
	#2: 157.16						

*Clinical information and CSF data are described for this patient; the patients with neurodegenerative diseases (bvFTD, FTD-MND, PSP, CBS, and AD) and the CTR subjects. Patient #1: the first hospitalization, #2: the present hospitalization. The data represent median ± standard error (S.E.)*.

**Figure 2:**
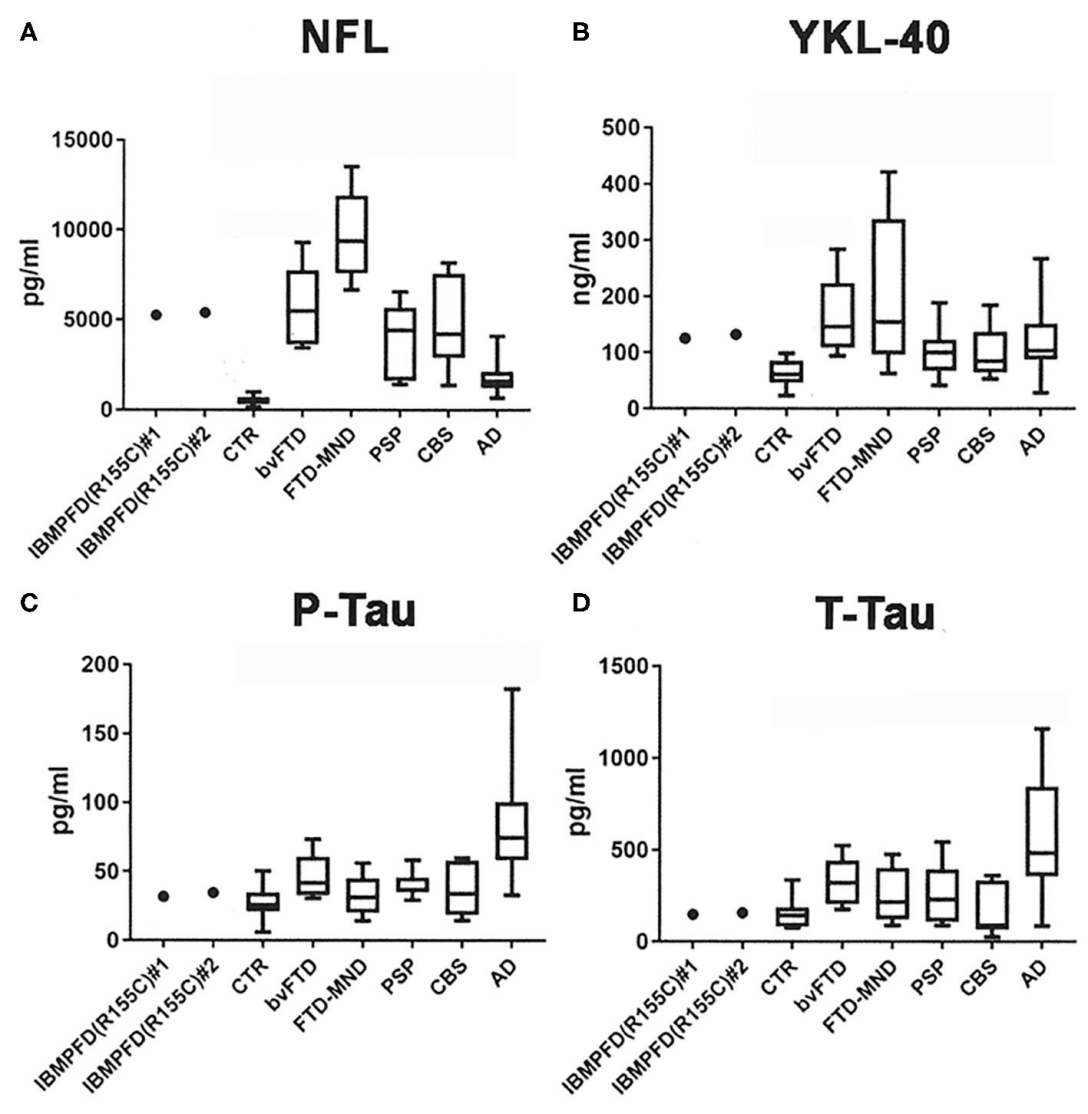
CSF analyses of NFL, YKL-40, phosphorylated tau 181 (P-Tau), and total human tau (T-Tau). **(A)** Both CSF NFL levels in the patient (#1: the first hospitalization and #2: the present hospitalization) were higher than those in CTR and lower than those in FTD-MND; further, the CSF NFL levels in bvFTD, PSP, CBS, and AD were higher than those in CTR. **(B)** Both CSF YKL-40 levels in the patient (#1 and #2) were higher than those in CTR; moreover, CSF YKL-40 levels in FTD-MND, bvFTD, PSP, and AD were higher than those in CTR. **(C)** CSF P-Tau levels in AD were higher than those in CTR and in neurodegenerative diseases including the patient (#1 and #2). **(D)** CSF T-Tau levels in AD were higher than those in CTR and other neurodegenerative diseases including the patient (#1 and #2). Bars in each graph present mean data.

The NFL CSF levels (pg/ml) in the patient were comparable at the two measurement points separated by 13 years (#1: the first puncture and #2: the second puncture). Both NFL CSF levels in the patient (#1: 5255.24 and #2: 5394.98) were higher than those in CTR individuals [452.93 ± 58.90; median ± standard error (S.E.)] and AD patients (1,531.70 ± 167.56), lower than those in FTD-MND patients (9,371.82 ± 1,134.69), and comparable to those in bvFTD (5,493.71 ± 814.18), PSP (4,413.78 ± 741.49), and CBS patients (4,217.29 ± 936.81; Figure 2A). The YKL-40 CSF levels (ng/ml) in the patient were comparable at the two times points (#1: the first time 125.03 and #2: the second time 132.41); furthermore, they were higher than those in CTR individuals (60.53 ± 5.43) and comparable to those in FTD-MND (154.39 ± 62.41), bvFTD (146.07 ± 25.87), PSP (99.94 ± 17.62), and AD (107.23± 10.26) (Figure 2B). The P-Tau CSF levels (pg/ml) in the patient (#1: 31.79 and #2: 34.72) were comparable to those in CTR individuals (25.52 ± 2.53), bvFTD (41.75 ± 6.10), FTD-MND (31.45 ± 6.87), PSP (36.60 ± 3.61), and CBS (33.93 ± 7.08), whereas the P-Tau levels (76.42 ± 7.73) in CSF of AD patients were higher than those in CTR individuals and patients with other neurodegenerative diseases (Figure 2C). The CSF levels of T-Tau (pg/ml) in the patient (#1: 148.60 and #2: 157.16) were comparable to those in CTR individuals (143.56 ± 16.07) and patients with other neurodegenerative diseases, whereas the CSF levels of T-Tau in AD patients (506.86 ± 71.60) were higher than those in CTR individuals (143.56 ± 16.07) and patients with other neurodegenerative diseases (Figure 2D). The CSF levels of both P-Tau and T-Tau in the patient were comparable to those in bvFTD, FTD-MND, PSP, and CBS patients (Figures 2C,D). These data are presented in Table 1.

## Discussion

IBMPFD is clinically characterized by adult-onset muscle weakness and atrophy, early-onset PDB, and frontotemporal dementia (FTD) (1, 2, 15). *VCP* is identified as the most predominant causative gene among IBMPFD patients, and the R155C mutation has been reported including Japanese ethnic background (2, 3, 9–14). VCP-related IBMPFD represents a unique class D subtype of the neurodegenerative diseases named TDP-43 proteinopathies with numerous ubiquitin-positive neuronal intranuclear inclusions and dystrophic neurites (31–33). Recently, CSF NFL has been investigated as a diagnostic marker of axonal neurodegeneration, especially ALS and frontotemporal lobar degeneration (FTLD) including bvFTD, PSP, CBS, and AD (5–7). Furthermore, YKL-40 has been identified as a CSF biomarker of glial neuroinflammation in ALS, FTLD, PSP, CBS, and AD (6–8).

This is the first report of a Japanese IBMPFD patient demonstrating higher and comparable levels, over 13 years, of the CSF biomarkers NFL and YKL-40 in an IBMPFD patient with a *VCP* mutation than in CTR individuals. Up to date, there is no other report but this case at least within the Japanese Consortium for Amyotrophic Lateral Sclerosis Research (JaCALS). The symptoms of this patient were not compatible with the typical ALS phenotype; however, the patient showed neurogenic changes in the EMG examination (data not shown) and neurogenic pathological changes in muscle biopsy. This patient is clinically expected to have poor prognosis, because his respiratory function has gradually deteriorated due to progressive general muscle weakness and atrophy due to IBMPFD. The patient will still require frequent aspirations of saliva and oxygen inhalation to support his respiration.

*VCP* mutations presumably lead to a dominant negative loss or alteration of VCP function culminating in impaired degradation of TDP-43 (34). Whereas IBMPFD is a multisystem proteinopathy (35), mutant VCP proteins are reportedly targets of autophagic-lysosomal degeneration, mitochondrial dysfunction, and ubiquitin–proteasome system disorders (36). A limitation of this study is the fact that only one patient of IBMPFD with a *VCP* mutation was included, which impeded statistical analyses for the other neurological diseases and CTR groups. NFL and YKL-40 levels were not compared in blood samples among the patient, noncarriers, and asymptomatic carriers with a *VCP* mutation to prove the utility of blood biomarkers for IBMPFD.

Higher NFL and YKL-40 CSF levels in the IBMPFD patient with a *VCP* mutation may be related to both axonal neurodegeneration and glial neuroinflammation. The implicated multifaceted pathological mechanisms should be elucidated, which may allow the discovery of new therapeutic targets for the *VCP* gene and/or the VCP protein in IBMPFD.

## Data Availability Statement

All data generated or analyzed during this study are included in this published article.

## Ethics Statement

The studies involving human participants were reviewed and approved by this study was approved by the ethics committee of the Gunma University Hospital (Masaki Ikeda) and the Jobu Hospital for Respiratory Diseases (Takeo Kuwabara). The patients/participants provided their written informed consent to participate in this study.

## Consent for Publication

Written consent to publish the clinical information was obtained from the patient's family.

## Author Contributions

MI and TK collected the clinical data and interpreted the data, and MI wrote the manuscript. ET analyzed the genomic DNA from the patient's blood samples and CSF biomarkers from the patient's CSF. YF performed the pathological examinations and evaluated the results. TH and YT evaluated the neuroimaging information. HK, MF, KM, AS, KN, and TY discussed the clinical information in terms of neurological features. MI and YI performed the clinical data analysis and evaluated their specificity and neurological significance. All authors contributed to the article and approved the submitted version.

## Conflict of Interest

The authors declare that the research was conducted in the absence of any commercial or financial relationships that could be construed as a potential conflict of interest.

## References

[1] Kimonis VE Kovach MJ Waggoner B Leal S Salam A Rimer L Clinical and molecular studies in a unique family with autosomal dominant limb-girdle muscular dystrophy and Paget disease of bone. Genet Med. (2000) 2:232–41. 10.1097/00125817-200007000-00006 11252708 PMC6173187

[2] Kimonis V Inclusion body myopathy with paget disease of bone and/or frontotemporal Dementia. In: Adam MP Ardinger HH Pagon RA Wallace SE Bean LJH Stephens K, editors. GeneReviews®. Seattle, WA: University of Washington (2007). p. 1993–2019 (Updated September 12, 2019). 20301649

[3] Watts GD Wymer J Kovach MJ Mehta SG Mumm S Darvish D. Inclusion body myopathy associated with Paget disease of bone and frontotemporal dementia is caused by mutant valosin-containing protein. Nat Genet. (2004) 36:377–81. 10.1038/ng1332 15034582

[4] Norgren N Rosengren L Stigbrand T. Elevated neurofilament levels in neurological diseases. Brain Res. (2003) 987:25–31. 10.1016/S0006-8993(03)03219-0 14499942

[5] Scherling CS Hall T Berisha F Klepac K Karydas A Coppola G. Cerebrospinal fluid neurofilament concentration reflects disease severity in frontotemporal degeneration. Ann Neurol. (2014) 75:116–26. 10.1002/ana.24052 24242746 PMC4020786

[6] Illán-Gala I Alcolea D Montal V Dols-Icardo O Muñoz L de Luna N. CSF sAPPβ, YKL-40, and NfL along the ALS-FTD spectrum. Neurology. (2018) 91:e1619–28. 10.1212/WNL.0000000000006383 30291183

[7] Molinuevo JL Ayton S Batrla R Bednar MM Bittner T Cummings J. Current state of Alzheimer's fluid biomarkers. Acta Neuropathol. (2018) 136:821–53. 10.1007/s00401-018-1932-x 30488277 PMC6280827

[8] Bonneh-Barkay D Wang G Starkey A Hamilton RL Wiley CA. *In vivo* CHI3L1 (YKL-40) expression in astrocytes in acute and chronic neurological diseases. J Neuroinflammation. (2010) 7:34. 10.1186/1742-2094-7-34 20540736 PMC2892443

[9] Schröder R Watts GD Mehta SG Evert BO Broich P Fliessbach K. Mutant valosin-containing protein causes a novel type of frontotemporal dementia. Ann Neurol. (2005) 57:457–61. 10.1002/ana.20407 15732117

[10] Guyant-Maréchal L Laquerrière A Duyckaerts C Dumanchin C Bou J Dugny F. Valosin-containing protein gene mutations: clinical and neuropathologic features. Neurology. (2006) 67:644–51. 10.1212/01.wnl.0000225184.14578.d3 16790606

[11] Gidaro T Modoni A Sabatelli M Tasca G Broccolini A Mirabella M. An Italian family with inclusion-body myopathy and frontotemporal dementia due to mutation in the VCP gene. Muscle Nerve. (2008) 37:111–4. 10.1002/mus.20890 17763460

[12] Stojkovic T Hammouda el H Richard P López de Munain A Ruiz-Martinez J Camaño P. Clinical outcome in 19 French and Spanish patients with valosin-containing protein myopathy associated with Paget's disease of bone and frontotemporal dementia. Neuromuscul Disord. (2009) 19:316–23. 10.1016/j.nmd.2009.02.012 19364651

[13] Shi Z Hayashi YK Mitsuhashi S Goto K Kaneda D Choi YC. Characterization of the Asian myopathy patients with VCP mutations. Eur J Neurol. (2012) 19:501–9. 10.1111/j.1468-1331.2011.03575.x 22040362

[14] Surampalli A Gold BT Smith C Castellani RJ Khare M Yu H. A case report comparing clinical, imaging and neuropsychological assessment findings in twins discordant for the VCP p.R155C mutation. Neuromuscul Disord. (2015) 25:177–83. 10.1016/j.nmd.2014.10.003 25582679 PMC5591024

[15] Weihl CC Pestronk A Kimonis VE Valosin-containing protein disease: inclusion body myopathy with Paget's disease of the bone and fronto-temporal dementia. Neuromuscul Disord. (2009) 19:308–15. 10.1016/j.nmd.2009.01.009 19380227 PMC2859037

[16] Neary D Snowden JS Gustafson L Passant U Stuss D Black S. Frontotemporal lobar degeneration: a consensus on clinical diagnostic criteria. Neurology. (1998) 51:1546–54. 10.1212/WNL.51.6.1546 9855500

[17] Cairns NJ Bigio EH Mackenzie IR Neumann M Lee VM Hatanpaa KJ. Neuropathologic diagnostic and nosologic criteria for frontotemporal lobar degeneration: consensus of the Consortium for Frontotemporal Lobar Degeneration. Acta Neuropathol. (2007) 114:5–22. 10.1007/s00401-007-0237-2 17579875 PMC2827877

[18] Mackenzie IR Neumann M Bigio EH Cairns NJ Alafuzoff I Kril J. Nomenclature for neuropathologic subtypes of frontotemporal lobar degeneration: consensus recommendations. Acta Neuropathol. (2009) 117:15–8. 10.1007/s00401-008-0460-5 19015862 PMC2710877

[19] Rascovsky K Hodges JR Knopman D Mendez MF Kramer JH Neuhaus J. Sensitivity of revised diagnostic criteria for the behavioural variant of frontotemporal dementia. Brain. (2011) 134:2456–77. 10.1093/brain/awr179 21810890 PMC3170532

[20] Balasa M Gelpi E Martín I Antonell A Rey MJ Grau-Rivera O. Diagnostic accuracy of behavioral variant frontotemporal dementia consortium criteria (FTDC) in a clinicopathological cohort. Neuropathol Appl Neurobiol. (2015) 41:882–92. 10.1111/nan.12194 25381753

[21] Lashley T Rohrer JD Mead S Revesz T. Review: an update on clinical, genetic and pathological aspects of frontotemporal lobar degenerations. Neuropathol Appl Neurobiol. (2015) 41:858–81. 10.1111/nan.12250 26041104

[22] Litvan I Agid Y Calne D Campbell G Dubois B Duvoisin RC. Clinical research criteria for the diagnosis of progressive supranuclear palsy (Steele-Richardson-Olszewski syndrome): report of the NINDS-SPSP international workshop. Neurology. (1996) 47:1–9. 10.1212/WNL.47.1.1 8710059

[23] Williams DR Lees AJ. Progressive supranuclear palsy: clinicopathological concepts and diagnostic challenges. Lancet Neurol. (2009) 8:270–9. 10.1016/S1474-4422(09)70042-0 19233037

[24] Armstrong MJ Litvan I Lang AE Bak TH Bhatia KP Borroni B. Criteria for the diagnosis of corticobasal degeneration. Neurology. (2013) 80:496–503. 10.1212/WNL.0b013e31827f0fd1 23359374 PMC3590050

[25] McKhann GM Knopman DS Chertkow H Hyman BT Jack CR Jr Kawas CH The diagnosis of dementia due to Alzheimer's disease: recommendations from the National Institute on Aging-Alzheimer's Association workgroups on diagnostic guidelines for Alzheimer's disease. Alzheimers Dement. (2011) 7:263–9. 10.1016/j.jalz.2011.03.005 21514250 PMC3312024

[26] American Psychiatric Association Diagnostic and Statistical Manual of Mental Disorders Fifth Edtion (DSM-5). Arlington, VA: American Psychiatric Association (2013).

[27] Dubois B Feldman HH Jacova C Hampel H Molinuevo JL Blennow K. Advancing research diagnostic criteria for Alzheimer's disease: the IWG-2 criteria. Lancet Neurol. (2014) 13:614–29. 10.1016/S1474-4422(14)70090-0 24849862

[28] Vanmechelen E Vanderstichele H Davidsson P van Kerschaver E van der Perre B Sjögren M. Quantification of tau phosphorylated at threonine 181 in human cerebrospinal fluid: a sandwich ELISA with a synthetic phosphopeptide for standardization. Neurosci Lett. (2000) 285:49–52. 10.1016/S0304-3940(00)01036-3 10788705

[29] Ikeda M Tashiro Y Takai E Kurose S Fugami N Tsuda K. CSF levels of Aβ1-38/Aβ1-40/Aβ1-42 and (11)C PiB-PET studies in three clinical variants of primary progressive aphasia and Alzheimer's disease. Amyloid. (2014) 21:238–45. 10.3109/13506129.2014.949231 25139672

[30] Andreasen N Minthon L Davidsson P Vanmechelen E Vanderstichele H Winblad B. Evaluation of CSF-tau and CSF-Abeta42 as diagnostic markers for Alzheimer disease in clinical practice. Arch Neurol. (2001) 58:373–9. 10.1001/archneur.58.3.373 11255440

[31] Forman MS Mackenzie IR Cairns NJ Swanson E Boyer PJ Drachman DA. Novel ubiquitin neuropathology in frontotemporal dementia with valosin-containing protein gene mutations. J Neuropathol Exp Neurol. (2006) 65:571–81. 10.1097/00005072-200606000-00005 16783167

[32] Neumann M Mackenzie IR Cairns NJ Boyer PJ Markesbery WR Smith CD. TDP-43 in the ubiquitin pathology of frontotemporal dementia with VCP gene mutations. J Neuropathol Exp Neurol. (2007) 66:152–7. 10.1097/nen.0b013e31803020b9 17279000

[33] van der Zee J Pirici D Van Langenhove T Engelborghs S Vandenberghe R Hoffmann M. Clinical heterogeneity in 3 unrelated families linked to VCP p.Arg159His. Neurology. (2009) 73:626–32. 10.1212/WNL.0b013e3181b389d9 19704082

[34] Irwin DJ Cairns NJ Grossman M McMillan CT Lee EB Van Deerlin VM. Frontotemporal lobar degeneration: defining phenotypic diversity through personalized medicine. Acta Neuropathol. (2015) 129:469–91. 10.1007/s00401-014-1380-1 25549971 PMC4369168

[35] Benatar M Wuu J Fernandez C Weihl CC Katzen H Steele J. Motor neuron involvement in multisystem proteinopathy: implications for ALS. Neurology. (2013) 80:1874–80. 10.1212/WNL.0b013e3182929fc3 23635965 PMC3908355

[36] Bayraktar O Oral O Kocaturk NM Akkoc Y Eberhart K Kosar A. IBMPFD Disease-causing mutant VCP/p97 proteins are targets of autophagic-lysosomal degradation. PLoS One. (2016) 11:e0164864. 10.1371/journal.pone.0164864 27768726 PMC5074563

